# Acknowledgement to Reviewers of *Insects* in 2019

**DOI:** 10.3390/insects11020072

**Published:** 2020-01-21

**Authors:** Insects Editorial Office

**Affiliations:** MDPI, St. Alban-Anlage 66, 4052 Basel, Switzerland

The editorial team greatly appreciates the reviewers who have dedicated their considerable time and expertise to the journal’s rigorous editorial process over the past 12 months, regardless of whether the papers are finally published or not. In 2019, a total of 481 papers were published in the journal, with a median time to first decision of 17 days and a median time from submission to publication of 44 days. The editors would like to express their sincere gratitude to the following reviewers for their generous contribution in 2019:
Abbott, JohnLopes, Alberto Jorge OliveiraAbro, Ghulam HussainLópez, AlejandroAcevedo, Flor EdithLopez Lastra, ClaudiaAdams, ChrisLópez-López, AlejandroAdamski, ZbigniewLorenzi, Maria-CristinaAddesso, KarlaLoudon, CatherineAdler, Peter H.Lövei, GáborAhmed, SaveerLu, ZhaozhiAhmed, Muhammad Z.Lubawy, JanAi, XiaLuce-Fedrow, AlisonAkino, ToshiharuŁukowski, AdrianAlbertini, AliceLynn, GeoffreyAlburaki, MohamedMa, ChenAlcolea, Pedro J.MacLean, Heidi J.Alford, LucyMadeira, Filipe NogueiraAllan, SandraMaekawa, KiyotoAllen, Margaret L.Magura, TiborAllen, Warwick J.Malacrinò, AntoninoAlves e Silva, Thiago LuizMalaquias, José BrunoAmarasekare, Kaushalya G.Mancini, EmilianoAmini, ShahrouzMandrioli, MauroAmiri, EsmaeilManicardi, Gian CarloAmorim, IsabelMankad, AditiAnastasaki, EiriniMankin, Richard W.Anderbrant, OlleMankowski, MarkAndersen, Jeremy C.Mann, Rajinder S.Anderson, Kirk E.Mans, BenAndo, IstvanMansour, RamziAndré, Marcos RogérioMansourian, SuzanAndreadis, StefanosMäntylä, ElinaAnfora, GianfrancoMantzoukas, SpiridonAnton, SylviaMarchant, RichardAntonatos, SpyridonMarcus, JeffreyAnzaldo, SalvatoreMargaritopoulos, John T.Aoki, ShigeyukiMarques, Maria RitaAppel, Arthur G.Marshall, Katie E.Apperson, Charles S.Marsico, TravisArias, Elizabeth T.Martemyanov, ViatcheslavArmstrong, Philip M.Martens, AndreasArnó, JuditMartin, StephenArthur, FrankMartinek, PetrAscrizzi, RobertaMartínez, Luis CarlosAtanasova, Daniela I.Martín-Hernández, RaquelAthanasiou, ChristosMartini, XavierAvalos, ArianMasetti, AntonioAvanesyan, AlinaMassimino Cocuzza, Giuseppe ErosAvarguès-Weber, AuroreMasuya, HayatoAvtzis, Dimitrios N.Mathiopoulos, Kostas D.Ayayee, Paul AkwetteyMatsumoto, YasuhikoBaba, Yuki G.Matsumura, YokoBadillo-Vargas, IsmaelMavridis, KonstantinosBagnoli, BrunoMay, MichaelBahar, Md HabibullahMazur, MiloszBaker, TomMazzeo, GaetanaBakovic, VidMazzi, DominiqueBaldini, FrancescoMazzoni, EmanueleBallardini, MarcoMazzoni, ValerioBara, JeffreyMbata, GeorgeBaracchi, DavidMcAfee, AlisonBarbosa, Bruno CorrêaMcCallum, RobynBarletta Ferreira, Ana BeatrizMcCravy, KennethBarman, ApurbaMcCreadie, JohnBarman, Apurba K.McCullough, Erin L.Barratt, BarbaraMcGhee, PeterBarrett, Bruce A.McNamara, LouiseBasnet, SanjayMcNeil, JeremyBau Macià, JosepMcNeill, CorraineBavaresco, LuigiMcRoberts, NeilBawin, ThomasMeasham, Penelope F.Baxter, Simon WadeMedrzycki, PiotrBeaurepaire, AlexisMehlhorn, HeinzBecher, Paul G.Meikle, WilliamBecker, NorbertMenezes, Irwin Rose AlencarBeers, ElizabethMenocal, OctavioBehle, Robert WarrenMenzel, RandolfBelcari, AntonioMercader, RodrigoBeliën, TimMermudes, Jose Ricardo MirasBellini, RoméoMerritt, DavidBenedict, Mark Q.Metz, Bradley N.Benìtez, EmilioMeuti, MeganBenowitz, Kyle M.Meyer, Wallace M.Berberich, Gabriele M.Meyer-Rochow, Victor BennoBeresford, DavidMezger, DirkBernardo, UmbertoMihai-Leonard, DudumanBerson, JacobMihalca, Andrei DanielBeyenbach, Klause W.Milanesio, DanieleBianchi, GiuliaMillar, Jocelyn G. Bignell, DavidMiller, ChristineBiondi, AntonioMiller-Struttmann, NicoleBiron, David GeorgesMills, Mary KatherineBlaauw, Brett R.Milonas, Panagiotis G.Blacquière, TjeerdMilosavljević, IvanBlair, CarolMishra, DushyantBlatt, Suzanne E.Mitchell, Forrest L.Bocak, LadislavMitchell, RobertBogdanowicz, Steven M.Mitrovic, Milana S.Bogusch, PetrMittapelly, PriyankaBohbot, JonathanMiyatake, TakahisaBohman, BjörnMogren, Christina L.Boieiro, MarioMolaei, GoudarzBorges, Paulo A.V.Montañez Martinez, RaulBorsuk, GrzegorzMontoya-Lerma, JamesBos, Nick Peter MariaMorales-Hojas, RamiroBoscardin, JardelMoran, NancyBosco, DomenicoMori, NicolaBosso, LucianoMori, BoydBowen, RichardMorrison, William R.Bradshaw, TerenceMorrison, Lloyd W.Breeuwer, HansMorrison, RobBriem, FelixMorrone, JuanBrown, SamuelMorrow, JenniferBrown, KatherineMorse, Douglass H.Brown, SebeMorse, GeoffBrownbridge, MichaelMota-Sanchez, DavidBrożek, JolantaMottern, Jason L.Brückner, AdrianMoulton, John KevinBrunet, Bryan M.T.Mound, Laurence A.Brutscher, LauraMoura, Luciano De AzevedoBryant, BartMousseau, TimothyBuchanan, AmandaMouton, LaurenceBulmer, Mark S.Müller, CarolineBurdfield-Steel, Emily R.Mullins, Aaron J.Burgio, GiovanniMuona, JyrkiBurian, StevenMusgrave, IanBurkett-Cadena, NathanMustafaf, GhazalaBurks, CharlesMuszyński, ArturBurrell, Brian B.Mutinelli, FrancoByrd, BrianNachappa, PunyaByrne, FrankNadel, HannahCabirol, AmélieNagalingam, KumaranCabrero-Sañudo, Francisco J.Nagata, ShinjiCaimano, MelissaNair, ShakuCalatayud, Paul-andréNalepa, Christine A.Caldara, RobertoNarayanasamy, SureshCalkins, TravisNardi, FrancescoCameron, DanielNardi, James B.Cammack, Jonathan A.Narouei-Khandan, HosseinCampbell, Alistair JohnNarouei-Khandan, Hossein A.Campbell, Corey L.Nash, David RichardCapbell, BrittanyNault, Brian A.Cardé, Ring T.Navarro, ShlomoCarrière, YvesNaviglio, DanieleCarvalho, DaniloNedvěd, OldřichCarvalho, Maria OtiliaNegron, JoseCastrejón-Gómez, Víctor RogelioNel, AndreCaterino, MichaelNice, Chris C.Cavalieri, VincenzoNieh, JamesChakrabarti, PriyadarshiniNielsen, Vance G.Chamorro, LourdesNielsen, Anne L.Chapman, Eric G.Nielsen-Leroux, ChristinaChapman, NadineNiño, Elina LastroChapman, Nadine C.Nishi, OumiChappell, Thomas M.Nishide, YudaiChaves, Luis FernandoNishino, HiroshiCheng, KenNishiwaki, HisashiChertemps, ThomasNjoroge, Anastasia W.Chevalier, MathieuNobre, Tânia MesquitaChitimia-Dobler, LidiaNofemela, RobertChou, HsuanNogami, MakotoChouvenc, ThomasNottingham, Louis B.Christiaens, OlivierNovak, LajosChrysoula, TananakiNtalli, Nikoletta G.Chuang, Ting-WuNugnes, FrancescoClarke, AnthonyNumata, HideharuClement, CristinaNunes, Francis Morais FrancoClemes , Danon CardosoNunes, LinaClifton, Eric H.Nyabuga, FranklinCocco, ArturoObonyo, DennisCochard, PrécilliaObrycki, John J.Colgan, Thomas J.Odemer, RichardCollatz, JanaOi, FaithCollett, Thomas S.Oi, David H.Collignon, Robert MaxwellOi, CintiaCollins, William R.O’Neal, ScottConti, BarbaraO’Neal, MatthewCook, GerickeOonincx, DennisCook, StevenOpachaloemphan, ComzitCoombes, CandiceOrtiz, AntonioCooper, RobinOsada, YoheiCooper, PaulOsborn, RachelCopren, Kirsten A.Ostiguy, NancyCordeiro, Erick M.G.Otero-Bravo, AlejandroCortet, JérômeOtti, OliverCosta-Da-Silva, André LuisOtuka, AkiraCover, Matthew R.Paddock, ChristopherCranston, PeterPallarés, SusanaCremer, SylviaPalmer, AlanCrickmore, NeilPalmeri, VincenzoCrossley, Michael S.Pandey, Atul KumarCrozatier-Borde, MichelePaoletti, MaurizioCull, BenjaminPape, ThomasCulliney, Thomas W.Paploski, IgorCunha, Hélida Ferreira DaPappas, MariaCuny, MaximilienPark, EugeneCuthbertson, Andrew G.S.Park, SangwookCzaczkes, Tomer JosephPark, YoonseongDa Silva, PedroPark, Hyun-WooDaane, Kent M.Patel, HardikkumarDacher, MatthieuPatrick, MarjorieDai, Shu-MeiPauchet, YannickDai, WuPaulson, DennisDajoz, IsabellePavela, RomanDamos, Petros T.Pechal, JenniferDaniels, JaretPekas, ApostolosDarbro, JonathanPeñalver, AinaraDavydenko, KaterynaPennacchio, FabrizioDawson, ErikaPentzold, StefanDe Souza, Danival JoséPenz, CarlaDe Souza, AlessandraPereira-Peixoto, Maria-HelenaDe Souza Loureiro, ElisângelaPerera, OmaththageDearden, PeterPerkin, LindseyDeisig, NinaPerry, Kayla I.Dekker, TeunPessoa, Luis Gustavo AmorimDel-Claro, KleberPetillon, JulienDell’Agli, MarioPetrakis, Panos V.Delsinne, ThibautPfliegler, Walter P.Démares, Fabien J.Phillips, Thomas W.Denecke, ShanePicanço, Ana Luísa CodernizDenton, Jerod S.Picciotti, UgoDepa, ŁukaszPickett, Charles H.Des Marteaux, LaurenPierce, Jane BreenDi Luca, MarcoPiersanti, SilvanaDi Palma, AntonellaPietri, Jose E.Di Pasquale, GarancePimentel, Ida ChapavalDiaz, CarolinaPiñero, JaimeDickerson, AndrewPinto, Maria AliceDillman, AdlerPirk, Christian W.W.Dillman, Adler R.Pizzolotto, RobertoDilts, TomPoh, Karen C.Dindo, Maria LuisaPoinar, GeorgeDionisio, GiuseppePolidori, CarloDitrich, TomášPondeville, EmilieDjoudi, El AzizPons, XavierDoak, Daniel F.Pope, TomDohmen-Vereijssen, JessicaPorcelli, FrancescoDolan, BenjaminPoulsen, MichaelDolezal, Adam G.Pozsgai, GaborDong, KePozzebon, AlbertoDonkersley, PhilipPramod, SreepriyaDonly, CamPrasad, AbhishekDonnell, David M.Prijić, AnetaDoorenweerd, CamielPrisley, SteveDorin, AlanPritt, BobbiDötterl, StefanPrunuske, AmyDoublet, VincentQualls, Whitney A.Doums, ClaudieQuarrell, StephenDowd, PatrickQuerner, PascalDrees, ClaudiaQuesada-Moraga, EnriqueDu, ZhentingQuinonez, Fausto ValenzuelaDuan, JianRadek, AulickDufour, Bernard P.Raimundi, ErikcsenDunphy, Michael J.Rajarapu, Swapna PriyaDupuis, JulianRajeswaran, JagadeesanDupuis, Julian R.Ranger, Christopher M.Duvic, BernardRashed, ArashDweck, Hany K.M.Rassati, DavideDyson, Paul J.Ray, AnnEberhard, Monika J.B.Raymond, BenEdwards, Marten JohnRazinger, JakaEgizi, AndreaReding, MichaelEinspanier, RalfReeves, LawrenceEizaguirre, MatildeRehman, Junaid UrElek, ZoltánReif, Kathryn E.Elghobashi-Meinhardt, NadiaRen, YonglinEliopoulos, Panagiotis A.Rendon, DalilaElleuche, SkanderRenkema, Justin M.Elsensohn, Johanna E. Renthal, RobertEndersby, NancyReznik, Jacqueline E.Endo, HarukaRhainds, MarcEnkerli, JürgRibeiro, JoséEntling, WiebkeRibeiro, Rita Cláudia CardosoErb, UweRicigliano, Vincent A.Erler, SilvioRicupero, MicheleErnst, UliRiddick, Eric WellingtonErram, DineshRiedel, AlexEtzler, Frank E.Riehle, MichaelEvenden, MayaRiudavets, JordiEvergetis, EpameinondasRivera-Pérez, CrisalejandraFahrbach, Susan E.Roberts, PhillipFaleiro, Maria LeonorRoberts, JoeFardisi, Mahsa N.Robinson, SamuelFauconnier, Marie LaureRobinson, KarlFavia, GuidoRobinson, Willard S.Feldhaar, HeikeRodriguero, MarcelaFelicioli, AntonioRodriguez, RafaelFeng, HonglinRodriguez Arrieta, JuanitaFernanda, ColombariRodriguez-Andres, JulioFernando, HataRodríguez-González, ÁlvaroFerrer, AurélieRodríguez-Rojas, AlexandroFiedler, KonradRodriguez-Saona, CesarFields, PaulRoellig, DawnFilipiak, MichałRoggero, AngelaFinke, Mark D.Rohlfs, MarkoFischer, DoreenRomani, RobertoFitches, ElaineRonderos, DavidFleming, Daniel E.Rondoni, GabrieleFLORIS, IgnazioRonga, DomenicoFochetti, RomoloRosell, Rosemarie C.Fontana, PaoloRosengaus, RebecaForneck, AstridRosenheim, Jay A.Fornoff, FelixRoss, PerranForsgren, EvaRossaro, BrunoFoust, Richard D.Rostislav, ZemekFoxi, CiprianoRoth, SteffenFrancati, SantoloRouhier, Matthew F.Francikowski, JacekRouttu, JarkkoFrank, Erik T.Rowland, KimberlyFriedrich, TheodorRoyer, TomFrooninckx, LotteRoyer, Jane E.Fuentealba, AlvaroRoy-zokan, Eileen M.Fukami, TadashiRubene, DianaGabrieli, PaoloRuberson, JohnGabryś, BeataRubiolo, PatriziaGadhave, KiranRückert, ClaudiaGaines-Day, Hannah R.Rugman-Jones, Paul F.Galbraith, Sara M.Ruiu, LucaGalizi, RobertoRuiz De Escudero, InigoGamboa, MaribetRuiz-Ruano, Francisco J.Gangloff-Kaufmann, Jody L.Rumbos, ChristosGanjisaffar, FatemehSagili, RameshGarcia, Flávio Roberto MelloSaigusa, KiyoshiGarcia Del Pino, FernandoSakamoto, Joyce M.Garrido-Jurado, InmaculadaSakurai, AkihikoGavin, MarySalanţă, Liana ClaudiaGebiola, MarcoSaloña-Bordas, Marta I.Geib, Scott M.Saquet, AdrianoGendrin, MathildeSaul-Gershenz, Leslie S.Gerken, Alison R.Schatton, AdrianaGezon, ZakScheff, Deanna S.Ghezzi, AlfredoScheffrahn, Rudolf H.Ghosh, SubirSchmidt, RebeccaGiamperi, LauraSchmidt, JasonGiatropoulos, AthanassiosSchmidt, TomGile, GillianSchmidt-Jeffris, RebeccaGiles, Kristopher L.Scholtens, Brian G.Gillett, Conrad P.D.T.Schultheiss, PatrickGioti, AnastasiaSciarretta, AndreaGiovenazzo, PierreScully, ErinGiunti, GiuliaSequeira, AndreaGloag, RosSeshadri, ArathiGnanvossou, DesireSeymoure, Brett M.Goblirsch, Michael J.Sgolastra, FabioGoddard, Matthew R.Shapiro, ArthurGodschalx, Adrienne L.Shapiro, ArthurGolick, DougShapiro Ilan, DavidGomez-Marco, FrancescSharma, AnamikaGomez-Moracho, TamaraShokal, UpasanaGomis-Cebolla, JoaquinShreeve, TimGondhalekar, Ameya D.Shrestha, ManiGonella, ElenaShrestha, PrachandGonthier, DavidSilk, PeterGonzález-Zamora, José EnriqueSimões, Zilá Luz PaulinoGotoh, HirokiSimone-Finstrom, Michael D.Grabenweger, GiselherSirot, LauraGrace, J. KennethSkaldina, OksanaGrapputo, AlessandroSkorokhod, OleksiiGrasso, GianvitoSkuhrovec, JiriGreenfield, MichaelSmartt, Chelsea T. Greenslade, PenelopeSmith, RyanGregorc, AlešSmith, Hugh A.Gries, GerhardSmith, LincolnGripenberg, SofiaSmodiš Škerl, MajaGross, AaronSnow, JonathanGross, JürgenSojka, DanielGryzińska, Magdalena MariaSokoloski, KevinGrzywacz, AndrzejSolensky, MichelleGu, LiuqiSolovyev, NikolayGuastella, DevidSommaggio, DanieleGuenat, SolèneSoper, Fiona M.Guerra, Patrick AnthonySpinelli, FrancescoGuerra-Grenier, EricSponsler, DouglasGui, Shun-HuaSrinivasan, RamasamyGuidolin, AlineStahl, Judith M.Gunter, NicoleStark, John D.Gupta, KuldeepSteen, TomokoGut, LarryStefanoff, PawelHa, Ngoc SanStejskal, VaclavHaack, RobertStelinski, Lukasz L.Haase, AlbrechtSterzyńska, MariaHaddi, KhalidStewart, AdamHaelewaters, DannyStockan, JenniHahn, Philip G.Stocker, AnnHalbert, Susan E.Stockton, DaraHall, DavidStout, Michael JosephHambäck, Peter A.Strauss, JohannesHan, Yeon S.Streinzer, MartinHan, Yeon SooStrunov, AntonHanly, Joseph J.Studebaker, Glenn E.Hano, ChristopheStyrsky, John D.Hansen, AllisonSubirana, JuanHanson, Anthony A.Sugahara, RyoheiHarbison, Justin E.Suh, EunhoHarmon-Threatt, Alexandra N. Suiter, Daniel R.Hartke, TamaraSunagar, KartikHarvey, Bret C.Sung, I-HsinHaseeb, MuhammadSutherland, Andrew M.Haselton, Aaron T.Suzuki, TakeshiHatt, SéverinSwanson, Brook O.Hatting, JustinSweeney, JonHayashi, YoshinobuSwitzer, CallinHayes, R. AndrewSzczepaniec, AdaHeadrick, David H.Szőcs, GáborHeckenhauer, JacquelineTabata, JunHeerman, MatthewTagu, DenisVan Helden, Maarten Tait, CheyenneHellemans, SimonTakatsuka, JunHemerik, LiaTakeda, MakioHenderson, GreggTalamas, ElijahHentley, William T.Talley, JustinHernandéz Mateo, LauraTamborindeguy, CeciliaHernández-Rodríguez, CésarTanaka, KazuhiroHerrin, DavidTanaka, ToshiharuHerz, AnnetteTang, PeianHespenheide, HenryTarasco, EustachioHeterick, Brian E.Tarone, AaronHiggins, Robert J.Taszakowski, ArturHirose, EuichiTatsuta, HarukiHoffmann, Klaus H.Tatsuya, KatoHoffmann, ChristophTaylor, BrianHogsette, Jerome A.Taylor, David B.Hohenstein, Jessica D.Teets, Nicholas M.Holloway, PaulTetreau, GuillaumeHolmstrup, MartinTettamanti, GianlucaHolst, NielsThangamani, SaravananHołyńska-Iwan, IgaTheodoropoulos, ChristosHönig, VáclavThomas, Donald B.Honti, ViktorThomson, LindaHooks, CerrutiThorn, SimonHorgan, FinbarrToft, SørenHotaling, ScottTokuda, GakuHottel, BenjaminTollerup, KrisHoward, Daniel R.Tomalia, DonaldHuang, Jen-PanTomanović, ŽeljkoHughes, GabrielTonina, LorenzoHull, J. JoeTonkyn, DaveHung, Keng-Lou JamesToševski, IvoHyodo, FujioTouchard, AxelIatsenko, IgorToussaint, Emmanuel F.A.Ibanez , FreddyTrager, Matthew D.Ibrahim, Sulaiman SadiTrager, James C.Iglesias, Lindsy E.Tragust, SimonIhle, Kate E.Traut, WaltherInagaki, HidetoshiTrdan, StanislavInui, YokoTrematerra, PasqualeIoannidis, PanagiotisTrewin, BrendanIoriatti, ClaudioTroczka, BartlomiejItakura, ShujiTrumble, John T.Jaffe, BenjaminTsagkarakis, AntoniosJaskuła, RadomirTsuchida, KojiJauker, FrankTucker, ErikaJean-Christophe, SimonTuda, MidoriJenkins, NinaTurney, ShaunJensen, KimTzin, VeredJeong, ChangyoonUnbehend, MelanieJesus, AvillaUnderwood, RobynJetter, KarenUnlu, IsikJian, FujiUrban, JulieJo, Yong HunUrbański, ArkadiuszJohnson, DonnVacas, SandraJohnson, Kelly S.Vagalinski, Boyan LyudmilovJohnson, Melissa A.Valdez, JoseJohnston, J. SpencerValenzuela-Gonzalez, IsabelJones, CarolValles, Steven M.Jones, IanVan Belleghem, StevenJones, DavidVan Der Burg, Karin R.L.Joseph, ShimatVanWeelden, Matthew T.Joshi, Neelendra K.Varloud, MarieJoyner, AndrewVasconcelos, Teresa MariaJucker, CostanzaVasilakis, NikosJudd, TimothyVea, Isabelle MifomJuhász, EditVelasco García, JosefaJung, Chol-HeeVenn, StephenJung, ChuleuiVenter, EduardKafle, LekhnathVerhulst, Niels O.Kageyama, DaisukeVigneron, AurelienKAINOH, YooichiVillalobos, Ethel M.Kalsi, MeghaVogelweith, FannyKamimura, YoshitakaVontas, JohnKang, DavidVotypka, JanKang, LeVuts, JozsefKang, SeokyoungWagoner, KairaKaramaouna, FilitsaWalgenbach, JamesKärhä, KalleWalker, ThomasKarlsson, Miriam FridaWaller, DeborahKazimírová, MariaWallinger, CorinnaKeasar, TamarWalton, William E.Keena, Melody A.Walton, Alexander R.Kehrli, PatrikWan, HuKelly, Richard S.Wang, HaikouKennedy, PeterWang, XingengKhadempour, LilyWang, YanKhanipov, KamilWang, ZhengKijimoto, TeiyaWang, XiaoyiKim, Byung-SooWang, HaichuanKimura, Masahito T.Wari, DavidKirker, GrantWaterworth, Rebeccah AnneKishimoto-Yamada, KeikoWay, MoKiya, TaketoshiWebb, Cameron E.Klepzig, Kier D.Webb, CameronKlitting, RaphaëlleWebb, CameronKlubertanz, Tom H.Webster, ThomasKnisley, Charles BarryWeeks, EmmaKnyshov, AlexanderWeiner, SusanKobori, YouichiWeintraub, Phyllis G.Kocarek, PetrWenninger, ErikKoehler, PhilipWesner, JeffKomives, TamasWest, Natalie M.Konagaya, TatsuroWetterer, James K.Konopka, Joanna K.Whittington, AndrewKooij, Pepijn W.Whyard, SteveKowalczewski, PrzemysławWickham, JacobKoyama, TakashiWiggins, GregKrams, IndrikisWilde, JerzyKrauel, JenniferWilhelm, GerthaKrell, Rayda K.Williams, TrevorKrewenka, Kristin M.Wilson, HoustonKrishnan, NatrajWilson, CliveKristensen, MichaelWilson, MichaelKrogh, Paul HenningWilson, HoustonKuhar, TomWitzgall, PeterKulma, MartinWong, Adam C.N.Kumar, PradeepWorischka, SusanneKumar, VivekWorthen, Wade B.Kuo, Yen-WenWright, DenisKuraishi, TakayukiWright, Mark G.Kuramitsu, KazumuWyckhuys, KrisKurtti, TimothyXu, JianKurze, ChristophXu, BaohuaKwak, Mackenzie L.Xue, Rui-DeLa Spina, MichelangeloYagound, BorisLabbé, Roselyne M.Yamaguchi, Lydia FumikoLahiri, SriyankaYan, JiayueLalander, CeciliaYan, HuaLancaster, JillYanagawa, AyaLara, RickyYang, Chin-Cheng ScottyLavista Llanos, SofíaYang, XiangbingLeather, Simon RobertYanoviak, Stephen P.Lecocq, AntoineYao, JianxiuLee, Kwang-ZinYasui, HiroeLee, Chow-YangYee, Wee L.Lees, Rosemary SusanYerabolu, Jayasudhan ReddyLeftwich, PhilipYokoi, KakeruLegal, LucYoshida, ShigetoLehmann, PhilippYoshioka, AkiraLehnert, MatthewYoshizawa, KazunoriLeigh, Brittany A.Zahniser, JamesLester, PhilZalucki, MyronLewis, Leslie C.Zattara, Eduardo E.Li, Hou-FengZehner, RichardLiao, Ling-HsiuZgomba, Marija F.Libbrecht, RomainZha, ChenLichtstein, DavidZhan, GuopingLill, John T.Zhang, TingLiu, JianfengZhang, Qing-HeLiu, HoupingZhang, SufangLiu, WenZhao, ChaoyangLiu, LeiZhu, LiLo Verde, GabriellaZhu, FangLong, Tristan A.F.Zhu, LuchangLoni, Augusto


